# Serological Survey of Retrovirus and Coronavirus Infections, including SARS-CoV-2, in Rural Stray Cats in The Netherlands, 2020–2022

**DOI:** 10.3390/v15071531

**Published:** 2023-07-12

**Authors:** Mirjam B. H. M. Duijvestijn, Nancy N. M. P. Schuurman, Johannes C. M. Vernooij, Michelle A. J. M. van Leeuwen, Berend-Jan Bosch, Judith M. A. van den Brand, Jaap A. Wagenaar, Frank J. M. van Kuppeveld, Herman F. Egberink, Josanne H. Verhagen

**Affiliations:** 1 Clinical Infectiology, Division of Infectious Diseases and Immunology, Department of Biomolecular Health Sciences, Faculty of Veterinary Medicine, Utrecht University, Yalelaan 1, 3584 CL Utrecht, The Netherlands; 2 Section of Virology, Division of Infectious Diseases and Immunology, Department of Biomolecular Health Sciences, Faculty of Veterinary Medicine, Utrecht University, Yalelaan 1, 3584 CL Utrecht, The Netherlands; 3 Division of Farm Animal Health, Department of Population Health Sciences, Faculty of Veterinary Medicine, Utrecht University, Yalelaan 7, 3584 CL Utrecht, The Netherlands; 4 Stray Cat Foundation Netherlands, Zuidzijdsedijk 90, 3264 LJ Hoeksche Waard, The Netherlands; 5 Division of Pathology, Department of Biomolecular Health Sciences, Faculty of Veterinary Medicine, Utrecht University, Yalelaan 1, 3584 CL Utrecht, The Netherlands

**Keywords:** seroprevalence, serology, feline, feline aids, viral infections, diagnosis, companion animals, zoonosis, coronavirus disease-19 (COVID-19), feral cats, trap neuter return and care (TNRC)

## Abstract

Stray cats can host (zoonotic) viral pathogens and act as a source of infection for domestic cats or humans. In this cross-sectional (sero)prevalence study, sera from 580 stray cats living in 56 different cat groups in rural areas in The Netherlands were collected from October 2020 to July 2022. These were used to investigate the prevalence of the cat-specific feline leukemia virus (FeLV, n = 580), the seroprevalence of the cat-specific feline viruses feline immunodeficiency virus (FIV, n = 580) and feline coronavirus (FCoV, n = 407), and the zoonotic virus severe acute respiratory coronavirus-2 (SARS-CoV-2, n = 407) using enzyme-linked immunosorbent assays (ELISAs). ELISA-positive results were confirmed using Western blot (FIV) or pseudovirus neutralization test (SARS-CoV-2). The FIV seroprevalence was 5.0% (95% CI (Confidence Interval) 3.4–7.1) and ranged from 0–19.0% among groups. FIV-specific antibodies were more often detected in male cats, cats ≥ 3 years and cats with reported health problems. No FeLV-positive cats were found (95% CI 0.0–0.6). The FCoV seroprevalence was 33.7% (95% CI 29.1–38.5) and ranged from 4.7–85.7% among groups. FCoV-specific antibodies were more often detected in cats ≥ 3 years, cats with reported health problems and cats living in industrial areas or countryside residences compared to cats living at holiday parks or campsites. SARS-CoV-2 antibodies against the subunit 1 (S1) and receptor binding domain (RBD) protein were detected in 2.7% (95% CI 1.4–4.8) of stray cats, but sera were negative in the pseudovirus neutralization test and therefore were considered SARS-CoV-2 suspected. Our findings suggest that rural stray cats in The Netherlands can be a source of FIV and FCoV, indicating a potential risk for transmission to other cats, while the risk for FeLV is low. However, suspected SARS-CoV-2 infections in these cats were uncommon. We found no evidence of SARS-CoV-2 cat-to-cat spread in the studied stray cat groups and consider the likelihood of spillover to humans as low.

## 1. Introduction

Cats can harbor a variety of (zoonotic) pathogens. The prevalence of these pathogens in stray cats is expected to be higher compared to domestic cats because stray cats may interact with a variety of other cats and with prey, while they are not treated or vaccinated to reduce disease occurrence [1]. A minimum of 135,000 stray cats and 3.1 million domestic cats were estimated to be present in The Netherlands in 2020 [2,3]. Stray cats are free-ranging cats that can either be domestic cats gone astray or feral unsocialized cats born in the wild [4,5]. These cats live in low or high cat-density groups varying from 1–2500 cats per km^2^ with a highly variable home range (0.0025–2.5 km^2^) [6]. The availability of food is an important determinant of the cat density and size of stray cat groups [1,5,6]. Cat group size, cat density and home range are determinants for the number of possible cat-to-cat contacts and, thus, for the possibility of pathogen exchange [5,6,7].

Most studies on stray cat groups focus on stray cats living in urban environments [7,8,9]. Urban stray cats may have more cat contacts outside their cat group than cats that live in (more isolated) rural stray cat groups. Rural stray cat groups have distinct habitats (e.g., holiday parks, campsites, industrial areas, farms) and living conditions within that habitat (e.g., availability and type of food, presence of human contact) compared to urban stray cat groups. Both urban and rural stray cats can interact with domestic free-roaming cats [6]. The prevalence of infectious diseases within stray cat groups depends on various factors, including transmission route and frequency and type of feline contact (not only among cats within the group but also between stray cats and free-roaming domestic cats) [10,11]. Moreover, variables such as age, sex and immune status can affect susceptibility to infection and/or disease as well as pathogen transmission within these cat groups [7,11,12,13,14]. Given that host and population-related factors contribute to pathogen prevalence, differences in pathogen (sero)prevalence among stray cat groups may be expected but remain largely understudied.

Stray cats can act as a pathogen source, from which spillover toward domestic cats or humans can occur. The seroprevalence of zoonotic pathogens in stray cats, such as *Chlamydia felis* (8%) [8], *Coxiella burnetii* (37–41%) [8,15] and *Toxoplasma gondii* (42–70%) [8,9,10], reflects their frequent exposure to these pathogens. In addition, zoonotic pathogens, such as *Leishmania infantum, Toxocara cati, Giardia duodenalis* and *Bartonella henselae,* have been detected in stray cats [9,10,12,16,17,18,19,20,21,22,23]. Co-infections with immunosuppressive viruses can increase stray cat susceptibility to infection with (zoonotic) pathogens [9]. For instance, feline immunodeficiency virus (FIV)-positive stray cats more often had antibodies against *T. gondii* and had higher titers of *T. gondii*-specific antibodies compared to FIV-negative stray cats [9]. Although pathogen exchange between stray cats and humans via direct contact may be limited, transmission may occur via feeding, roaming garbage, wastewater or excrement, particularly when stray cat home ranges overlap locations where humans reside [8,24]. In addition, a geospatial analysis of feral cats in Richmond, United States of America (USA), showed that territories of 81.5% of feral cats overlap with zones that were frequented by children, who are particularly vulnerable to infection with specific zoonotic pathogens [24]. Pathogen monitoring programs in stray cats can be used to investigate past or current infections with pathogens that can pose a risk to humans and domestic cats. Moreover, these programs can be used to monitor the health status of stray cats and monitor the effect of health improvement measures in stray cats [11,25].

The retroviruses FIV and FeLV can cause persistent infections in cats, resulting in severe immunosuppression [10,26,27]. Aggressive interaction among cats is the main transmission route of FIV, while FeLV is transmitted via saliva during grooming or sharing food and water bowls [5,27,28,29]. Persistent FIV-infected cats are typically identified based on FIV-specific antibody detection [30,31], while persistent FeLV-infected cats can be identified based on FeLV antigen detection [27,29,30]. In Europe, FIV-specific antibodies and FeLV antigen have been detected in domestic cats [27,32] as well as in urban stray cats [9,10,11,12,21,23,27,33,34]. Hence, retroviruses are present in domestic cats and urban stray cats in Europe, and contact between domestic cats and stray cats may facilitate virus transmission.

Feline coronavirus (FCoV), transmitted via the fecal–oral route [35,36], is a common feline pathogen that can cause a range of clinical symptoms from mild diarrhea to life-threatening feline infectious peritonitis (FIP) [35,37,38]. The presence of FCoV-specific antibodies reflects previous exposure, and an elevated FCoV-specific antibody titer is positively correlated with the presence of FCoV in feces. In Europe, a high FCoV seroprevalence (up to 80%) has been associated with high-density domestic cat groups [35,36,38,39], while lower seroprevalences were detected in urban stray cats (Birmingham in the United Kingdom, 22.4%; Milan in Italy, 39%) [12,13]. FIP was most likely the cause of death in 13/186 stray cats in Milan, Italy [40]. Thus, FCoV infections may be less common in stray cats than in domestic cats in Europe but can result in FCoV-associated diseases such as FIP [39,41].

Early in the SARS-CoV-2 pandemic, it was shown that domestic cats could become infected upon exposure to their SARS-CoV-2-infected owners [42,43,44,45,46,47]. The SARS-CoV-2 seroprevalence in domestic cats varies in different feline cohorts, with up to 52% (25/48) seropositivity found in cats living with SARS-CoV-2-infected owners [43,48,49,50]. Most of these SARS-CoV-2-infected cats remained subclinical or showed mild respiratory clinical signs [42,43]. In experimental settings, intranasal SARS-CoV-2-inoculated cats showed nasal or oropharyngeal virus shedding 1–10 days post infection [51,52,53,54] and showed cat-to-cat transmission [51,52,53,55]. The reproduction number R_0_ was calculated to be between 2.3 and 3.3 based on SARS-CoV-2 experimental and natural transmission studies. This suggests that SARS-CoV-2 has the potential to spread within groups of cats [51,52,53,55]. The SARS-CoV-2 seroprevalence in European stray cats was found to be low (0.0–2.3%) [17,18,19,20,23,56,57], but rural stray cats have not been specifically targeted.

In Europe, studies on the (sero)prevalence of FIV, FeLV, FCoV and SARS-CoV-2 focus on stray cats in urban areas; however, little is known about (sero)prevalence in rural stray cats [9,11,12,13,19,20,21,22,23,57]. Viral infections in stray cats (urban or rural) in The Netherlands remain understudied. In this study, we investigated the ((co-)sero)prevalence of cat-specific (FIV, FeLV, FCoV) and zoonotic (SARS-CoV-2) viruses in stray cat groups in rural areas in The Netherlands and explored the association of the (sero)prevalence with age, sex, health status and location type.

## 2. Materials and Methods

### 2.1. Study Population, Data Collection and Sampling

Stray cats (n = 580) were sampled at rural locations in The Netherlands between 5 October 2020 and 15 July 2022 (excluding 15 December 2020 to 12 April 2021 due to COVID-19 restrictive measures) during Trap Neuter Return and Care (TNRC) actions conducted by the Stray Cat Foundation Netherlands (SCFN). Locations were chosen based on notifications of the public, reporting overpopulation and/or nuisance of a group of at least 10 stray cats per location in rural areas (i.e., areas with an address density of less than 1000 per km^2^ [58]). TNRC programs are considered an effective and ethical alternative to culling stray cats and are well suited for infectious disease serosurveillance because sampling of cats can be easily implemented during anesthesia [25].

Locations were situated at least 2.5 km apart, and thus cat groups were considered as separate without overlapping home ranges [2,6]. Each location was categorized into dairy farm, industrial area, countryside residence, holiday park or campsite or nature reserve based on the information of the SCFN. Next, each location was categorized into two cat density categories as previously described (divided into moderate to high (50–100 cats per km^2^) or low density (<50 cats per km^2^) groups) [6,7], based on descriptions provided by the SCFN. Information provided by the SCFN on availability of indoor access and potential access to shared litter boxes and number of human contacts (low (1–5 people) to high (>10 people)) was used to characterize location types. The characteristics of these location types are described in Table S1.

Cats were caught using bait traps. Cats that were previously neutered (recognized as ear tipped) or had an owner (recognized by the presence of an identification chip) were excluded from sampling, resulting in a single sample per cat. Upon capture, cats were handled in a mobile veterinary clinic (KATOPIA^®^) or at a local veterinary practice. Cats were sedated using ketamine, dexmedetomidine and buprenorphine. After clinical evaluation, the cats were neutered/spayed, treated for parasites, vaccinated, ear tipped and finally, blood samples were collected. Surgery was not performed on cats < 1 kilogram (kg); therefore, cats < 1 kg were excluded from the study. Age was estimated using clearly defined criteria on teeth, eyes and general condition and was categorized as a binary variable (under 3 years or ≥3 years of age [14]); this ensured that each category contained at least 100 cats. The variable “sex” in our study consisted of intact males and females. The variable “health status” was defined as “apparently healthy” or “unhealthy” based on the evaluation of clinical abnormalities described by the veterinarian of the SCFN. A diversity of clinical abnormalities was found in these cats, such as wounds, abscesses, tumors, feline upper respiratory tract disease, gingivitis, dental problems, cachexia, heavy lice infestation, ocular problems, etc. Cats with these abnormalities were coded as “unhealthy”. The sample size per location was categorized as ≤10 samples and >10 samples per location.

Blood samples were taken from the jugular or cephalic vein and collected in a gel-and-clot activator tube (Greiner 108 Bio-One, Kremsmunster, Austria). A maximum of three ml was collected, which was within the limit volume for single sampling in animal studies (5.7% of circulating blood volume for cats of 1 kg) [59]. Blood samples were transported to the laboratory at room temperature within two days of sampling. Upon arrival at the laboratory, samples were stored at 4 °C and processed within 24 h. Serum tubes were centrifuged for 10 min, and serum was heat-inactivated for 30 min at 56 °C, aliquoted and stored at −20 °C prior to analysis. In case of low serum volume (<200 µL), priority was given to detection of FIV-specific antibodies and FeLV antigen, followed by detection of FCoV- and SARS-CoV-2-specific antibodies. We chose the pathogens FIV, FeLV and FcoV given their different transmission routes and given the immunosuppressive effect of FIV and FeLV, while SARS-CoV-2 was included as sampling occurred during the COVID-19 pandemic and little was known at that time about SARS-CoV-2 susceptibility and cat-to-cat transmission under field conditions.

### 2.2. Ethics

In concordance with directive 2010/63 of the European Parliament, no ethical approval was needed to perform this type of study. Blood samples were taken for veterinary diagnostic purposes for FIV-specific antibodies and FeLV detection, and results were reported to the veterinarian of the SCFN. Surplus serum was used to detect coronavirus-specific antibodies.

### 2.3. Virus Detection

To detect FeLV antigen in the cat sera, the commercially available ViraCHEK FELV ELISA targeting the P27 antigen of FeLV was used according to the manufacturer’s instructions (Zoetis Inc., Kalamazoo, MI, USA).

### 2.4. Antibody Detection

To detect FIV-specific antibodies, three different commercially available ELISAs (PetChek FIV-specific antibody test kit (IDEXX bioanalytics, Westbrook, CT, USA), ViraCHEK FIV (Synbiotics Europe, Lyon, France), MegaELISA FIV (MEGACOR Diagnostik GmbH, Hoerbranz, Austria)) were used according to the manufacturer’s instructions based on the availability of the tests. The performance of two of the ELISAs (PetChek and ViraCHEK) was compared previously and considered comparable [30]. According to the manufacturer (unpublished data), the Mega ELISA FIV sensitivity was 98.8%, and the specificity was 97.9% when 184 feline sera were tested and compared to a commercial ELISA. Serum samples that tested positive for FIV-specific antibodies in the ELISA were tested in the gold standard Western blot, as described by Egberink et al. (1991) [60] with minor alterations: we used purified recombinant FIV proteins (CA, KSU3, TM2 (part of GP41)) and lysed whole virus (for P24). Samples were regarded as FIV-specific antibody-positive if ≥2/4 bands were detected in the Western blot [60].

To detect FCoV-specific antibodies, an in-house-developed indirect ELISA based on the S1 subunit of the FCoV type I spike protein was used, as in Zhao et al. [61]. In short, FCoV S1 protein was used to coat ELISA plates, and serum samples (1:50 diluted) were added. After incubation and washing, horseradish peroxidase (HRP)-conjugated goat anti-cat antibody and subsequent TMB (3,3′5,5′-tetramethylbenzidine) were added to the plate to visualize FCoV-specific antibodies. The optical density (OD) was measured at 450 nm. An SPF cat serum was used as negative control, and 1:600 diluted ascites from an FCoV-experimentally-infected SPF cat were used as positive control on all plates. The FCoV ELISA used detects both FCoV type I—as well as FCoV type II—specific antibodies [61].

To detect SARS-CoV-2-specific antibodies, two different indirect ELISAs, one based on the S1 subunit of the SARS-CoV-2 spike protein and one based on the receptor binding domain (RBD) of the SARS-CoV-2 S1 subunit of the ancestral SARS-CoV-2 Wuhan-Hu-1 strain were used, as previously described [49]. The ELISA procedure was the same as for FCoV-specific antibody detection and used streptavidin-tagged SARS-CoV-2 S1 or SARS-CoV-2 RBD proteins produced as in Wang et al. (2020) [62]. SPF cat serum was used as a negative control, and a SARS-CoV-2 seropositive domestic cat serum (year 2020, S1-ELISA positive, RNB-ELISA positive, pseudo-VNT positive, 1:50 diluted [49]) was used as a positive control on all plates.

Serum samples were considered positive in the FCoV or SARS-CoV-2 ELISA if the optical density (OD) value was above the cut-off value, as described by Zhao et al. (2019) and Zhao et al. (2021) [49,61]. All sera were tested in duplo and were retested (in duplo) if the relative standard deviation (SD) of the two samples was >20%. To minimize inter-assay variation, SARS-CoV-2 ELISA S1 and RBD-positive serum samples were repeated when sufficient volume (>100 µL) was available in duplo on the same plate.

As a complementary test, SARS-CoV-2 S1- or SARS-CoV-2 RBD ELISA-positive samples and a selection of 20 SARS-CoV-2 ELISA-negative samples were tested for the presence of neutralizing antibodies in a pseudovirus neutralization test (pseudo-VNT) using luciferase-encoding vesicular swine virus (VSV) particles pseudo-typed with S1-protein of SARS-CoV-2 (Wuhan-Hu-1 strain) as in Wang et al. (2020) [62]. Pseudo-VNT assays and assays using wildtype SARS-CoV-2 showed good correlation for the detection of neutralizing SARS-CoV-2-specific antibodies in humans, while the pseudo-VNT can be performed under BSL-2 containment conditions [63]. Briefly, two-fold dilutions of serum samples were incubated with equal volumes of SARS-CoV2-S1-luciferase encoded VSV, and after incubation, the mixture was seeded on Vero E6 cells. After incubation, the cells were washed and lysed. Luciferin was added to the lysate, and the relative luminescence units (RLU) were measured. The neutralization titers were determined at 50% reduction of luciferase activity, with pseudo-VNT ≥ 16 considered positive, as described previously [49]. Serum samples with an ELISA OD value higher than the cut-off for both S1 and RBD protein that were negative in the pseudo-VNT were considered “suspected”, as defined in Zhao et al. (2021) [49].

To compare the sensitivity of the SARS-CoV-2 pseudo-VNT used here with results published previously, four pseudo-VNT-positive cat sera from previous studies [43,49] (i.e., one mink-exposed and three household-exposed) were retested in our pseudo-VNT [43,49] and gave similar or two-fold higher neutralizing antibody titers. Based on these findings, we conclude that our pseudo-VNT was comparable in performance to the assay that was earlier described (data not shown) [43,49]. In addition, to detect low concentrations of neutralizing antibodies in the stray cat sera, we tested one SARS-CoV-2 S1 and RBD ELISA negative, one SARS-CoV-2 S1 ELISA-positive but RBD-negative, and one SARS-CoV-2 S1 and RBD ELIS-positive sample in the pseudo-VNT, starting at 1:4 dilution [43].

As an additional validation for the SARS-CoV-2 pseudo-VNT test results, 26 stray cat samples were analyzed in a commercial multispecies blocking ELISA, according to the manufacturer’s instructions (SARS-CoV-2 Surrogate Virus Neutralization Test Kit, GenScript Biotech, Rijswijk, Netherlands) [64]. Binding of HRP-labeled-RBD to the ACE2 receptor protein coated on the pate can be blocked by SARS-CoV-2 RBD neutralizing antibodies in the serum, if present. This test showed excellent concordance with the gold standard plaque reduction neutralization test (PRNT_90_) [65]. The 26 samples used for this additional validation were SARS-CoV-2 ELISA-negative (n = 8), SARS-CoV-2 RBD ELISA-positive (n = 4), SARS-CoV-2 S1 ELISA-positive (n = 8) or positive in both the SARS-CoV-2 S1 ELISA and RBD ELISA (n = 6).

### 2.5. Data Analysis

Data analysis and graphic presentation were performed using SPSS (IBM SPSS Statistics for Windows, Version 28.0, IBM Corp. Released 2021, Armonk, NY, USA) and GraphPad (GraphPadPrism version 9.3.1 for Windows, GraphPad Software, San Diego, CA, USA). To map the sample locations, Datawrapper was used [66]. To estimate the sample size for an apparent prevalence of 1% for SARS-CoV-2 (desired precision of 1%, 95% confidence interval (CI) with an estimated population size of 135.000), a sample size calculation was performed [2,67]. Based on this analysis, the sample size required a minimum of 381 cats. To calculate the (sero)prevalence (including 95% CI) of viral infections in stray cats in The Netherlands, the Exact Binominal test was used. To measure the degree of association between ELISA OD values of SARS-CoV2-S1 and SARS-CoV-2 RBD, the Spearman rank correlation test was used. To analyze potential associations of age, sex, health status, location type, sample size (≤10 or >10 per location) or co-seropositivity based on the presence of virus-specific antibodies and/or antigen, a univariable risk analysis was performed and the χ2 or Fisher’s exact test was used. Associations were expressed as relative risk (RR) providing 95% confidence intervals, and statistical significance was set to *p* < 0.05. To calculate the RR in categories with zero events, 0.5 was added to all frequencies involved (Firth’s correction), as in Ghebremariam et al., 2018 [68]. When a univariable risk factor analysis was performed on the categorical variable “location type”, the most frequent category (i.e., dairy farm) was used as a reference category. The convenience sampling scheme used in this study resulted in a biased study population (e.g., towards younger cats); therefore, we chose to describe various characteristics of the population in relation to the serology. To determine the effect of the inclusion of locations with a small sample size (at least one cat per location) on the FIV, FCoV and SARS-CoV-2-suspected seroprevalence and strength of associations, results of the full data were compared with results of data selecting locations with at least 10 samples (thus excluding the locations with a small sample size). The seroprevalences and associations with the different variables in this subset of data showed the same trend (Tables S2 and S3).

## 3. Results

### 3.1. Characteristics of the Study Population and Samples

**Figure 1:**
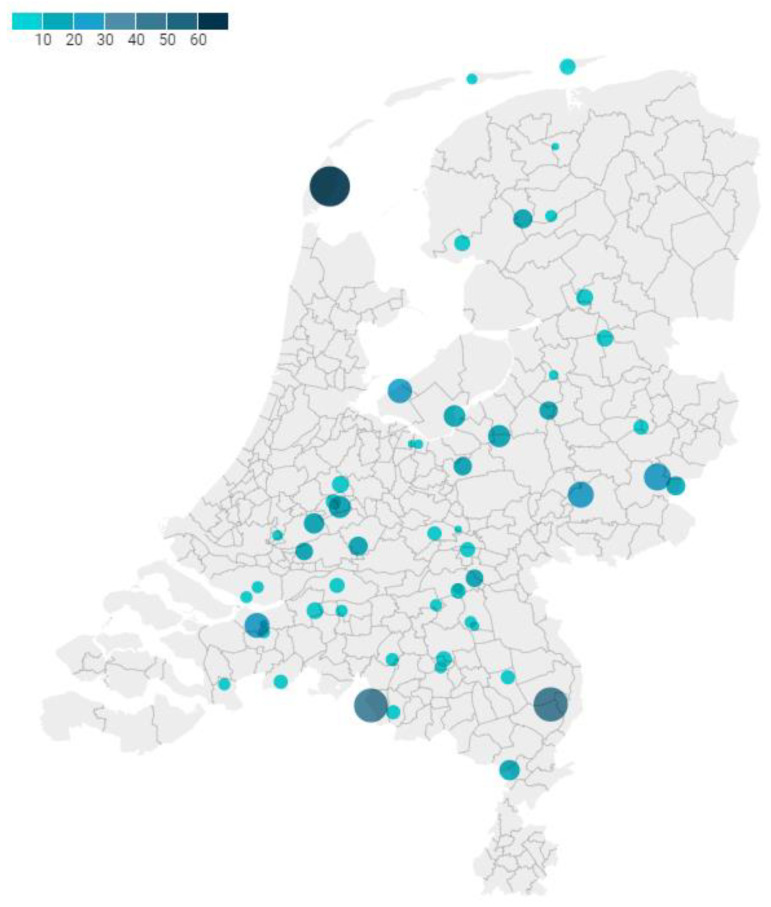
Spatial distribution of rural stray cat (n = 580) sample locations (n = 56) in The Netherlands sampled from 2020 to 2022. The diameter and color of the circle represent the number of cats sampled per location (smallest circle depicts one cat, largest circle depicts 62 cats).

Stray cat serum samples were collected from cat groups in 56 locations distributed across The Netherlands, as shown in Figure 1. The number of cats sampled per location ranged from 1 to 62 samples (sample size median 16; IQR 8–43) (Figure 1).

Most locations (n = 39/56) were sampled once. Locations with a larger cat population were sampled more than once. In total, 91/580 (15.7%) cats were sampled in 2020, 295/580 (50.9%) cats were sampled in 2021 and 194/580 (33.4%) cats were sampled in 2022. Sampling events were mainly carried out from March–June (288/580, 49.7%) and October–November (147/580, 25.3%). This was due to low volunteer availability and other unfavorable trapping conditions during the summer and due to low temperatures and/or COVID-19 measures during the winter.

For the majority of samples, data on estimated age (n = 562/580), sex (n= 573/580) and health status (n = 516/580) were available. Location-specific information (i.e., postal code and habitat type) was available for 532/580 samples.

More female cats (320/573, 55.8%) than male cats (253/573, 44.2%) were sampled. The estimated age of 440/562 (78.3%) cats was under 3 years, and 122/562 (21.7%) cats were estimated to be 3 years or older. A total of 392/511 (76.0%) cats appeared healthy, and 122/511 (24.0%) cats appeared unhealthy. More than half of the cats were sampled at dairy farms (294/532, 55.3%), 32/532 (6.0%) at industrial areas, 78/532 (14.7%) at countryside residences, 78/532 (20.9%) at holiday parks or campsites and 17/532 (3.2%) in nature reserves. None of the stray cats lived at or near (1 km) a mink or poultry farm.

### 3.2. (Sero)prevalence Retroviruses and Coronaviruses in Stray Cats

All 580 samples (from 56 locations) were tested for both FIV-specific antibodies and FeLV antigen, and a subset of 407 samples (from 39 locations, sample-size median 21, IQR 9–43) were tested for FCoV- and SARS-CoV-2-specific antibodies. In 29/580 cats, FIV-specific antibodies were found in the ELISA test, of which 28/580 were confirmed by Western blot, resulting in a seroprevalence of 5.0% (95% CI: 3.4–7.1%). None of the 580 samples tested positive in the ELISA for FeLV antigen (95% CI: 0.0–0.6). Out of 407 samples that were tested for FCoV-specific antibodies, 137 (33.7%, 95% CI: 29.1–38.5%) were positive. A total of 27/407 samples had specific antibodies for the S1 protein of SARS-CoV-2 (Figure S2). In eleven of these samples (11/407), SARS-CoV-2 RBD-specific antibodies were detected, and these samples were considered suspected and were used to calculate the SARS-CoV-2 suspected seroprevalence of 2.7% (95% CI: 1.4–4.8%). In addition, nine samples reacted with the SARS-CoV-2 RBD protein but not with the SARS-CoV-2 S1 protein (Figure S2). The Spearman’s rank correlation coefficient (n = 407) between the ELISA OD values for SARS-CoV-2 S1 and SARS-CoV-2 RBD was 0.72 (95% CI: 0.67–0.76) (Figure S2). None of the samples that were positive in either the SARS-CoV-2 ELISA or SARS-CoV-2 RBD ELISA (36/407) and that were tested in the pseudo-VNT (35/55) showed SARS-CoV-2-neutralizing antibodies. The twenty samples that were negative in the SARS-CoV-2 ELISAs were also negative in the pseudo-VNT. One sample that was positive in both SARS-CoV-2 ELISA tests could not be tested in the pseudo-VNT because of low sample volume. The start serum dilution of 1:4 instead of 1:16 did not result in the detection of neutralizing antibodies. The 26 SARS-CoV-2 ELISA-positive and negative (and pseudo-VNT negative) stray cat sera that were tested in the surrogate-VNT as additional validation for the pseudo-VNT did not inhibit RBD-ACE-2 binding. Hence, we confirmed the absence of neutralizing antibodies in the SARS-CoV-2-suspected cat sera.

### 3.3. FIV Seroprevalence and Associations with Age, Sex, Health Status, Location Type, Sample Size and Seropositivity for Other Viruses

In Table 1, the strength of the association of FIV seropositivity with the possible risk factors or determinants of infection is presented. The overall FIV seroprevalence was 5.0%, and on 14/56 locations (25%), FIV seropositive samples were collected (Figure S1A). The FIV seroprevalence varied significantly per location (*p* = 0.017), ranging from 0% (0/43) to 19.0% (4/21) (Figure S1A).

Cats aged 3 years or older were 4.2 times more likely to be FIV-specific antibody-positive (*p* < 0.001) compared to cats that were estimated to be younger than 3 years. We did not find FIV-specific antibody-positive stray cats under 1 year of age. Male cats were 6.1 times more likely to be FIV-positive (*p* < 0.001) compared to female cats. Cats that were unhealthy were 4.9 times more likely to be FIV-positive (*p* < 0.001) compared to cats that appeared healthy. Reported health problems in unhealthy FIV-positive (14/124 FIV+) and unhealthy FIV-negative (110/124 FIV-) cats that could be associated with FIV were, e.g., wounds or abscesses (4/14 vs. 4/110 resp.), tumors (2/14 vs. 3/110 resp.) and severe gingivitis and dental problems (3/14 vs. 19/110 resp.). Due to severe clinical symptoms, 5/14 unhealthy FIV-positive cats and 5/110 unhealthy FIV-negative cats were euthanized. Cats that lived at holiday parks, campsites or nature reserves were less likely to be FIV-specific antibody-positive compared to cats that lived at dairy farms, industrial areas or countryside residences (*p* = 0.27). There was no association between FIV status and number of cats sampled at a location (≤ 10 or > 10) (*p* = 0.43). FIV status of the cats was not associated with FCoV seropositivity (*p* = 0.31) nor with SARS-CoV-2-suspected seropositivity (*p* = 0.43).

### 3.4. FCoV Seroprevalence and Associations with Age, Sex, Health Status, Location Type, Sample Size and Seropositivity for Other Viruses

In Table 2, the strength of the association of FCoV seropositivity with possible risk factors or determinants of infection is presented. The overall seroprevalence for FCoV was 33.7%, and in 25/39 sample locations (64%), cats with FCoV-specific antibodies were detected (Figure S1B). The seroprevalence for FCoV was significantly different per type of location (*p* < 0.001), ranging from 4.7% (2/43) to 85.7% (12/14) (Figure S1B).

Cats 3 years or older were 1.57 times more likely to be FCoV seropositive (*p* < 0.002) compared to cats younger than 3 years. No significant differences were detected in the presence of FCoV-specific antibodies between males and females (*p* = 0.093). The seroprevalence of FCoV in healthy cats was 1.8 times lower than the seroprevalence in unhealthy cats (*p* < 0.001). The clinical symptoms noted in the unhealthy FCoV-specific antibody-positive cats were not suggestive of FCoV-associated diseases, such as diarrhea or ascites [39]. Cats that lived in industrial areas or countryside residences were 1.73 and 1.42 times more likely to be FCoV seropositive compared to cats living at dairy farms (*p* = 0.018). Cats that lived at holiday parks or campsites were 0.83 times less likely to be FCoV-seropositive compared to cats that lived at dairy farms. No cats from a nature reserve were tested for FCoV-specific antibodies. There was no association between FCoV status and number of cats sampled at a location (≤10 or >10) (*p* = 0.252). There was no significant co-seropositivity for FCoV-positive cats and SARS-CoV-2-suspected cats (*p* = 0.079) and no association between FCoV-specific and SARS-CoV-2-specific ELISA OD values (Figure S3A,B).

### 3.5. SARS-CoV-2 Suspected Seropositivity and Associations with Age, Sex, Health Status, Location Type, Sample Size and Seropositivity for Other Viruses

In Table 3, the strength of the association of SARS-CoV-2-suspected seropositivity with possible risk factors or determinants of infection is presented. The overall seroprevalence of SARS-CoV-2-suspected seropositive cats was 2.7% (11/407), and these cats were found in 9/39 (23%) locations (Figure S1C). The prevalence of SARS-CoV-2-suspected cats was not significantly different per type of location (*p* = 0.53), and only one location had more than one SARS-CoV-2-suspected cat (3/11) (Figure S1C).

There were no significant associations in SARS-CoV-2-suspected cats with age (*p* = 0.62), sex (*p* = 0.76), health status (*p* = 0.31), location type (*p* = 0.91), sample size (*p* = 0.521) or co-seropositivity (*p* = 0.193).

## 4. Discussion

In this study, we investigated the potential role of rural stray cats in The Netherlands as a source of retroviruses and coronaviruses by exploring the (sero)prevalence of FIV, FCoV, FeLV and SARS-CoV-2. Significant differences in the presence of FIV-specific antibody-positive and FCoV-specific antibody-positive cats among rural stray cat groups were detected. Factors associated with an elevated (sero)prevalence were older age (FIV and FCoV), male sex (FIV) and unhealthy cats (FIV and FCoV). We found that location type was associated with FCoV seropositivity; cats sampled at industrial areas and countryside residences were FCoV seropositive more often compared to cats sampled at dairy farms, holiday parks or campsites. No FeLV-positive stray cats were detected. The SARS-CoV-2-suspected seroprevalence was low, and in 8/9 SARS-CoV-2-suspected locations, only a single SARS-CoV-2-suspected cat was detected.

The FIV, FeLV and FCoV (sero)prevalence, as detected in rural stray cats in this study, was compared with the (sero)prevalence in urban stray cats and domestic cats. Most (sero)prevalence studies sampled stray cats via TNRC programs, and therefore, the capture and sampling methods were considered comparable. Based on European studies on stray cats living in cities, FIV seroprevalence of 5.0% in this study was low compared to the FIV seroprevalence elsewhere in Europe 6.1–19.3% [9,10,11,12,21,23], and the FCoV seroprevalence of 33.7% in this study was similar to the FCoV seroprevalence elsewhere in Europe (Italy 39%, UK 22.4%) [12,13]. No FeLV-positive rural stray cats were found in this study, whereas elsewhere in Europe, in urban stray cats, the prevalence ranged from 0.7 to 7.1% [9,10,11,12,21,23,27]. Next, the (sero)prevalence of the rural stray cats of this study were compared to (sero)prevalence as reported previously in domestic cats in The Netherlands (or elsewhere in Europe if no data was available for The Netherlands). The FIV seroprevalence of 5% was comparable with the FIV seroprevalence in domestic cats in Germany (3.2%) [69]. The FCoV seroprevalence of 33.7% in this study was lower than in domestic cats sampled in The Netherlands in 2019 (78/137, 56.9%) [61]. No FeLV-positive rural stray cats were detected in this study, and the absence of FeLV infection was also shown in domestic cats in The Netherlands in 2019 (0/356, 0%) [27]. Not only were strong differences in seroprevalence shown to exist among different countries, but here large differences in seroprevalence were found among different stray cat groups, previously also described in Belgian urban stray cats (ranged from 7.9% to 30.5% in five locations) [11,70,71].

We found several determinants of infection or risk factors that can explain these location-specific seroprevalence differences. FIV-specific antibodies in rural stray cats in this study were more prevalent in intact tomcats and older stray cats, as was shown previously for urban stray cats [7,9,26,34]. The presence or absence of tomcats—and thus aggressive interactions facilitating FIV transmission—may explain why FIV is maintained in some rural stray cat groups but is absent in others. Furthermore, differences in social structures (e.g., familiar groups show less aggressive interactions) could account for differences in seroprevalence among cat groups, but information regarding social structures was not available. The elevated FIV seroprevalence in older cats in this study could be related to a prolonged period of exposure in older animals. The lack of FIV-persistent infected cats <1 year can be the result of the overall low seroprevalence, with only 5/320 female cats persistently infected. We found an elevated FIV seroprevalence in unhealthy cats, and these cats more often had wounds, abscesses or tumors (which can be related to FIV-associated disease) compared to FIV-negative unhealthy cats [26,27,28,41,72]. While it has been reported that FIV-infected domestic cats can have a normal life expectancy [26,41], we found that 5/14 FIV-positive unhealthy cats were euthanized due to severe untreatable health problems [26,27,28,41,72]. Lack of veterinary care and elevated exposure to pathogens may explain the impaired health status and reduced life expectancy of FIV-infected stray cats compared to domestic cats. We did not find a significant association between FIV seropositivity and (sero)positivity for the other viruses tested. In our dataset, unhealthy cats (regardless of FIV status) were found significantly more often in cats 3 years or older (45.5%) compared to cats younger than 3 years (18.1%) (*p* < 0.001). We, therefore, have to take into account that the association between FIV-seropositivity and health problems could be due to a confounding effect with age. We used three different commercial FIV ELISA antibody detection tests that we considered similar in performance. When the seroprevalence is low, as our results indicate, the positive predictive value of these commercial ELISAs may be low. False positive test results were, however, only found in 1/29 ELISA-positive cats.

In this study, an elevated FCoV seroprevalence was detected in cats sampled at industrial areas or countryside residences—where cats lived in moderate-to-high-density cat groups with indoor access and shared litterboxes—compared to cats from low-density cat groups sampled at holiday parks or campsites that had a strict outdoor life-style [41,72]. Transmission of FCoV occurs via fecal–oral contact, which, in domestic cats, is associated with an indoor life-style with shared litterboxes and high cat density [38,39,41,72,73]. In contrast, outdoor living or free-roaming cats bury their feces and avoid fecal contact, which may negatively affect FCoV transmission [35,39,41]. The elevated FCoV seroprevalence that was found in older cats in this study was also found in urban stray cats in the UK and could be related to a prolonged period of exposure in older animals [13]. FIP—a systemic infection that can develop as a result of FCoV infection—is a problem associated with high-density cat groups where FCoV circulates. We found an elevated FCoV seroprevalence in unhealthy stray cats in this study, but the reported health problems were not associated with FCoV-associated disease. It cannot be excluded that the association between FCoV seropositivity and health status was affected by the confounding variable age.

In contrast to earlier findings, in this study, SARS-CoV-2-specific antibodies detected by ELISA did not neutralize SARS-CoV-2 in the pseudo-VNT test. Discrepancies between ELISAs that detect binding antibodies, and assays that detect neutralizing antibodies, have been reported earlier [43,48,49,74,75] Several explanations for the absence of neutralizing antibodies were investigated. An explanation for the presence of binding antibodies in the absence of neutralizing antibodies could be that the time between SARS-CoV-2 exposure and sampling in this cross-sectional study was unknown, and waning immunity may have resulted in the absence of neutralizing antibodies, although SARS-CoV-2 neutralizing antibodies in naturally infected cats could be detected for at least 10 months post exposure [51,52,54,76]. Additionally, most SARS-CoV-2-infected cats show little or no clinical signs, and this can result in the induction of variable and low levels of antibodies [43,46,49,54]. During the SARS-CoV-2 pandemic, multiple antigenically different SARS-CoV-2 variants have been circulating with different levels of cross-reactivity. An infection with SARS-CoV-2 variants was considered but discarded as the explanation for the absence of neutralizing antibodies in SARS-CoV-2 S1 and RBD ELISA-positive cats in this study [77,78]. SARS-CoV-2 Alpha, Delta and Omicron BA.1 variants can infect cats under natural and experimental conditions [52,54,57,79] Infections in cats with either the Alpha or Delta variant induced antibodies that showed good cross-reactivity in ELISA, s-VNT, pseudo-VNT or PRNT using the ancestral SARS-CoV-2 strain [54,57,80,81,82]. In households with infected humans, human-to-cat spread of Omicron variants was markedly reduced when compared to human-to-cat spread of the Delta variant, suggesting a reduced susceptibility in cats for Omicron variants [83]. Omicron BA.1 experimental infected cats showed delayed and lower neutralizing antibody titers, which did not neutralize the ancestral SARS-CoV-2 strain [54]. Given this lack of cross-reactivity, sera from stray cats exposed to Omicron BA.1 could have reacted in the ELISA based on the ancestral SARS-CoV-2 variant and not in the more specific pseudo-VNT based on the ancestral SARS-CoV-2 variant. However, the SARS-CoV-2 ELISA-positive cats in this study were sampled during the Alpha and/or Delta wave (n = 8) in The Netherlands or during a period when the Delta variant was dominant and the Omicron BA.1 was in its early stage of emergence (n = 3). Based on the temporal spread of the SARS-CoV-2-ELISA-positive sera, exposure of stray cats to Omicron BA.1 and consequently low cross-reactivity with the ancestral SARS-CoV-2 strain as used in the pseudo-VNT can insufficiently explain the lack of neutralizing antibodies. Cross-reactivity between antibodies induced by other coronaviruses that might infect stray cats was considered another explanation for the SARS-CoV-2-ELISA-positive results in the absence of SARS-CoV-2-neutralizing antibodies [50,84,85]. In this study, there was no association between the ELISA OD values of SARS-CoV-2 S1/RBD and FCoV S1 (Figure S3A,B). Therefore, it seems unlikely that FCoV-cross-reactive antibodies account for SARS-CoV-2 ELISA-positive results in this study, which had been excluded during the validation of the assays [49]. In other studies, cross-reactivity between FCoV-specific antibodies and SARS-CoV-2 antigen had also been excluded in cats sampled since the SARS-CoV-2 pandemic [23,49,50,74,86]. In contrast to these studies, experimental FCoV-infected SPF cats showed cross-reactivity in a SARS-CoV-2 RBD-specific ELISA [85]. In addition, pre-pandemic sera from cats sampled in the USA tested positive in a SARS-CoV-2 RBD-specific ELISA [84]. We further considered cross-reactivity in the sera due to an infection with other coronaviruses, for instance, β-coronaviruses present in bats [87]. Cats regularly prey on bats, and this could lead to exposure and infection with these coronaviruses [88]. However, whether this could then result in cross-reactivity with SARS-CoV-2-antigens remains to be explored [87]. In conclusion, the detection of SARS-CoV-2 S1 and RBD-positive but non-neutralizing antibodies could not be explained completely, but poor immunogenicity of SARS-CoV-2 viruses in cats, waning immunity and variable cross-reactivity among different SARS-CoV-2 variants and other coronaviruses have been considered as explanatory factors. The low SARS-CoV-2 suspected seroprevalence (2.7%) in rural stray cats in this study was in concordance with a serological survey of shelter cats in The Netherlands in 2020 (where 2.1% cats [5/240] had SARS-CoV-2 ELISA S1 and RBD-specific antibodies, of which two (0.8%) also had neutralizing antibodies), and with other European surveys in urban stray cats [18,19,20,23,57,75]. This was in contrast to the higher seroprevalence observed in domestic cats in The Netherlands from households where owners were recently infected with SARS-CoV-2 (19.1%; 29/152) or randomly sampled at veterinary practices (6.4%; 9/140) [43]. Given that SARS-CoV-2-specific antibodies were detected in a single cat in 8/9 SARS-CoV-2-suspected rural stray cat groups, transmission among stray cats included in our study is unlikely, and humans (or domestic cats) are more likely to be the source of SARS-CoV-2 infection for stray cats than vice versa.

Pathogen monitoring programs in stray cats can be beneficial for human, domestic cat and stray cat health and can be part of a pandemic preparedness program for surveillance of emerging (zoonotic) viruses [89]. Targeted testing of stray cats—i.e., older, intact male, unhealthy cats—can be used to assess the presence of FIV infection in stray cat groups. The return or relocation of FIV-positive stray cats after neutralization, however, can spread the virus and disease. Interestingly, the removal of FIV-specific antibody-positive cats from Belgian urban stray cat groups resulted in a reduction of the FIV seroprevalence from 30% seropositivity to 13% seropositivity within 2 years [11]. Using TNRC in combination with FIV test-and-removal for over two decades, a reduction in FIV seroprevalence and an increase in stray cat welfare was found in Key Largo, USA [25]. In addition, the low FeLV prevalence in The Netherlands, also found in other countries in Western and Northern Europe, has been linked to successful FeLV test-and-removal programs in domestic and stray cats [27]. TNRC combined with FIV/FeLV on-the-spot testing and subsequent removal can therefore be considered an effective intervention that will result in a reduction of the stray cat population, an increase in stray cat health and welfare, and a reduced risk of retrovirus spread to free-roaming domestic cats. FCoV-infected stray cats can shed virus for at least two years [35,39], and high titers of FCoV-specific antibodies were more often found in cats that were active virus shedders [36]. Therefore, adoption into multi-cat households or rehoming to shelters of stray cats originating from particularly high-density cat groups (e.g., industrial areas or countryside residences) may facilitate FCoV spread.

## 5. Conclusions

In this study, we focused on rural stray cats as hosts of retroviruses and coronaviruses and found highly variable seroprevalences for FIV and FCoV among cat groups. Due to our sampling method not being random, we focused on univariable analyses to further explore these differences. Older, intact tomcats were more often FIV seropositive and can spread the virus (via fighting) to domestic cats. To reduce this risk, stray cat population control using trapping and neutering can be combined with targeted retrovirus testing. Given the occurrence of FIV-associated disease in FIV-infected stray cats, the return of FIV seropositive cats after neutering can be debated. We conclude that the likelihood of FeLV transmission between stray cats and free-roaming domestic cats, based on the low prevalence in both stray and domestic cats in The Netherlands, is low. We conclude that transmission—in particular of FCoV—between stray cats and domestic cats will mainly occur when stray cats are relocated to shelters or adopted into multi-cat households. The low SARS-CoV-2-suspected seroprevalence in rural stray cats, with mostly a single seropositive cat per cat group, suggests an absence of SARS-CoV-2 spread within rural stray cat groups and a negligible risk for cat-to-human transmission.

## Figures and Tables

**Table 1:** Strength of association (frequencies and relative risk) of FIV seropositivity with age, sex, health status, location type, sample size and co-seropositivity in n = 580 rural stray cats in The Netherlands from 2020 to 2022.

	Total ^#^		FIV Positive		FIV Negative				
Variable	Nr.	%	Nr.	%	Nr.	%	Relative Risk	95% CI *	*p*-Value
**Total**	580	100	29	5.0	551	95.0		3.4–7.1	
**Estimated age**									<0.001
**<3 years**	440	78.3	12	2.7	428	97.3	Ref		
**≥3 years**	122	21.7	14	11.5	108	88.5	4.2	2.0–8.9	
**Sex**									<0.001
**Female**	320	55.8	5	1.6	315	98.4	Ref		
**Male**	253	44.2	24	9.5	229	90.5	6.1	2.4–15.6	
**Health status**									<0.001
**Apparently**	392	76.0	9	2.3	383	97.7	Ref		
**healthy**									
**Unhealthy**	124	24.0	14	11.3	110	88.7	4.9	2.2–11.1	
**Location type**									0.27
**Dairy Farm**	294	55.3	17	5.8	277	94.2	Ref		
**Industrial area**	32	6.0	2	6.3	30	93.8	1.1	0.26–4.5	
**Countryside**	78	14.7	6	7.7	72	92.3	1.2	0.50–3.0	
**residence**									
**Holidayparc/** **campsite**	111	20.9	2	1.8	109	98.2	0.31	0.01–1.3	
**Nature reserve**	17	3.2	0	0.0	17	100	0.47	0.20–1.1	
**Sample size**									0.43
**≤10**	202	34.8	8	4.0	194	96.0	Ref		
**>10**	378	65.2	21	5.6	357	94.4	1.4	0.6–3.1	
**Co-seropositivity**									
**FeLV neg**	578	100.0	29	5.0	549	95.0			
**FeLV pos**	0	0.0	0	0.0	0				
**FCoV neg**	270	66.3	13	4.9	257	95.1	Ref		0.31
**FCoV pos**	137	33.7	10	7.2	127	92.8	1.5	0.68–3.4	
**SARS-CoV-2 neg**	396	97.3	23	5.8	373	94.2	Ref		0.43
**SARS-CoV-2**	11	2.7	0	0.0	11	100.0	0.70	0.30–1.7	
**suspected**									

FeLV: feline leukemiavirus detected using antigen-ELISA, FIV: feline immunodeficiency virus detected using antibody-ELISA, FCoV: feline coronavirus detected using antibody-ELISA, SARS-CoV-2 suspected: severe acute respiratory syndrome coronavirus-2-specific antibody-positive for the S1 subunit of the SARS-CoV-2 spike protein and the RBD receptor binding domain of the S1 subunit of SARS-CoV-2 detected using antibody ELISA, Nr: number, RR: relative risk, CI: confidence interval, Ref: reference category. * 95% CI Total represents the CI around the FIV seroprevalence, 95% CI for the determinants represents the CI around the relative risk. # Not all serum samples were accompanied by all metadata; therefore, the total numbers within a variable can be lower than N = 580.

**Table 2:** Strength of association (frequencies and relative risk) of FCoV seropositivity with age, sex, health status, location type, sample size and co-seropositivity in rural stray cats in The Netherlands from 2020 to 2022.

	Total ^#^		FCoV Positive		FCoV Negative				
Variable	Nr.	%	Nr.	%	Nr.	%	Relative Risk	95% CI *	*p*-Value
**Total**	407	100	137	33.7	270	66.3		29.1–38.5	
**Estimated age**									0.002
**<3 years**	304	75.4	90	29.6	214	70.4	Ref	Ref	
**≥3 years**	99	24.6	46	46.5	53	53.5	1.6	1.2–2.1	
**Sex**									0.093
**Female**	228	56.0	69	30.3	159	69.7	Ref	Ref	
**Male**	178	43.7	68	38.2	110	61.8	1.3	0.96–1.7	
**Health status**									<0.001
**Apparently** **healthy**	283	69.5	83	29.3	200	70.7	Ref	Ref	
**Unhealthy**	94	23.1	49	52.1	45	47.9	1.8	1.4–2.3	
**Location type**									0.018
**Dairy Farm**	202	54.2	65	32.2	137	67.8	Ref	Ref	
**Industrial area**	18	4.8	10	55.6	8	44.4	1.7	1.1–2.7	
**Countryside residence**	59	15.8	27	45.8	32	54.2	1.4	1.0–2.0	
**Holiday parc/** **campsite**	94	25.2	25	26.6	69	73.4	0.83	0.56–1.2	
**Nature reserve**	0	0	NA	NA	NA	NA	NA	NA	
**Sample size**									0.252
**≤10**	119	29.2	35	29.4	84	70.6	Ref		
**>10**	288	70.8	102	35.4	186	64.6	1.2	0.87–1.7	
**Co-seropositivity**									
**FeLV neg**	407	100.0	137	33.7	270	66.3			
**FeLV pos**	0	0.0	0	0.0	0	0			
**FIV neg**	384	94.3	127	33.1	257	66.9		Ref	
**FIV pos**	23	5.7	10	43.5	13	56.5	1.3	0.81–2.1	0.27
**SARS-CoV-2neg**	396	97.3	131	33.1	265	66.9		Ref	
**SARS-CoV-2 suspected**	11	2.7	6	54.5	5	45.5	1.7	0.94–2.9	0.079

FeLV: feline leukemiavirus detected using antigen-ELISA, FIV: feline immunodeficiency virus detected using antibody-ELISA, FCoV: feline coronavirus detected using antibody-ELISA, SARS-CoV-2 suspected: severe acute respiratory syndrome coronavirus-2-specific antibody-positive for the S1 subunit of the SARS-CoV-2 spike protein and the RBD receptor binding domain of the S1 subunit of SARS-CoV-2 detected using antibody ELISA. Nr: number, RR: relative risk, CI: confidence interval, Ref: reference category, NA: not applicable. * 95% CI Total represents the CI around the FCoV seroprevalence, 95% CI for the determinants represents the CI around the relative risk. # Not all serum samples were accompanied by all metadata; therefore, the total numbers within a variable can be lower than N = 407.

**Table 3:** Strength of association (frequencies and relative risk) of SARS-CoV-2-suspected seropositivity with age, sex, health status, location type, sample size and co-seropositivity in rural stray cats in The Netherlands from 2020 to 2022.

	Total ^#^		SARS-CoV-2 Suspected		SARS-CoV-2 Negative				
Variable	Nr.	%	Nr.	%	Nr.	%	Relative Risk	95% CI *	*p*-Value
**Total**	407	100	11	2.7	396	97.3		1.4–4.8	
**Estimated age**									0.62
**<3 years**	304	75.4	9	3.0	295	97.0	Ref		
**≥3 years**	99	24.6	2	2.0	97	98.0	0.68	0.15–3.1	
**Sex**									0.762
**Female**	228	56.0	7	3.1	221	96.9	Ref		
**Male**	178	43.7	4	2.2	174	97.8	0.73	0.22–2.5	
**Health status**									0.31
**Apparently** **healthy**	283	69.5	10	3.5	273	96.5	Ref		
**Unhealthy**	94	23.1	1	1.1	93	98.9	0.30	0.039–2.3	
**Location type**									0.91
**Dairy Farm**	202	54.2	7	3.5	195	96.5	Ref		
**Industrial area**	18	4.8	0	0.0	18	100	0.71	0.29–1.7	
**Countryside** **residence**	59	15.8	2	3.4	57	96.6	0.98	0.21–4.6	
**Holiday parc/** **campsite**	94	25.2	2	2.1	92	97.9	0.61	0.13–2.9	
**Nature reserve**	0	0	NA	NA	NA	NA	NA		
**Sample size**									0.52
**≤10**	119	29.2	2	1.7	117	98.3	Ref		
**>10**	288	70.8	9	3.1	279	96.9	1.8	0.40–8.4	
**Co-seropositivity**									
**FeLV neg**	407	100.0	0	0.0	396	100.0			
**FeLV pos**	0	0.0	0	0.0	0	0.0			
**FIV neg**	384	94.3	11	2.9	373	97.1	Ref		
**FIV pos**	23	5.7	0	0.0	23	100.0	0.70	0.29–1.7	0.43
**FCoV neg**	384	94.3	5	1.9	265	98.1	Ref		
**FCoV pos**	23	5.7	6	4.4	131	95.6	2.4	0.73–7.6	0.193

FeLV: feline leukemiavirus detected using antigen-ELISA, FIV: feline immunodeficiency virus detected using antibody-ELISA, FCoV: feline coronavirus detected using antibody-ELISA, SARS-CoV-2 suspected severe acute respiratory syndrome coronavirus-2-specific antibody-positive for the S1 subunit of the SARS-CoV-2 spike protein and the RBD receptor binding domain of the S1 subunit of SARS-CoV-2 detected using antibody ELISA. Nr: number, RR: relative risk, CI: confidence interval, Ref: reference category, NA: not applicable. * 95% CI Total represents the CI around the SARS-CoV-2-suspected seroprevalence, 95% CI for the determinants represents the CI around the relative risk. # Not all serum samples were accompanied by all metadata; therefore, the total numbers within a variable can be lower than N = 407.

## Data Availability

Data available upon request.

## Supplementary Materials

The following supporting information can be downloaded at: https://www.mdpi.com/article/10.3390/v15071531/s1, Figure S1: Number of seropositive and seronegative rural stray cats per sample location in The Netherlands, 2020–2022; Figure S2: Correlation between the ELISA optical density of SARS-CoV-2 RBD-specific antibodies and SARS-CoV-2 S1-specific antibodies in stray cat serum samples (n = 407) from The Netherlands, 2020–2022; Figure S3: Comparison of the ELISA optical density of FCoV-specific antibodies and SARS-CoV-2-specific-antibodies in stray cat serum samples from The Netherlands, 2020–2022; Table S1: Location type and characteristics of rural stray cat sample locations in The Netherlands, 2020–2022; Table S2: Strength of association (frequencies and relative risk) of FIV seropositivity with age, sex, health status, location type and co-seropositivity in rural stray cats sampled in The Netherlands from 2020–2022, based on locations where at least 10 cats were sampled per location; Table S3: Strength of association (frequencies and relative risk) of FCoV seropositivity with age, sex, health status, location type, and co-seropositivity in rural stray cats sampled in The Netherlands from 2020–2022, based on locations where at least 10 cats were sampled per location.
