# Supplementary material for: Serological Survey of Retrovirus and Coronavirus Infections, including SARS-CoV-2, in Rural Stray Cats in The Netherlands, 2020–2022
Source: Viruses. 2023 Jul 12;15(7):1531. doi: 10.3390/v15071531 (PMC10385588; doi:10.3390/v15071531)
Supplement: Supplementary file 1 [file viruses-15-01531-s001.zip › viruses-2451664-supplementary.pdf]

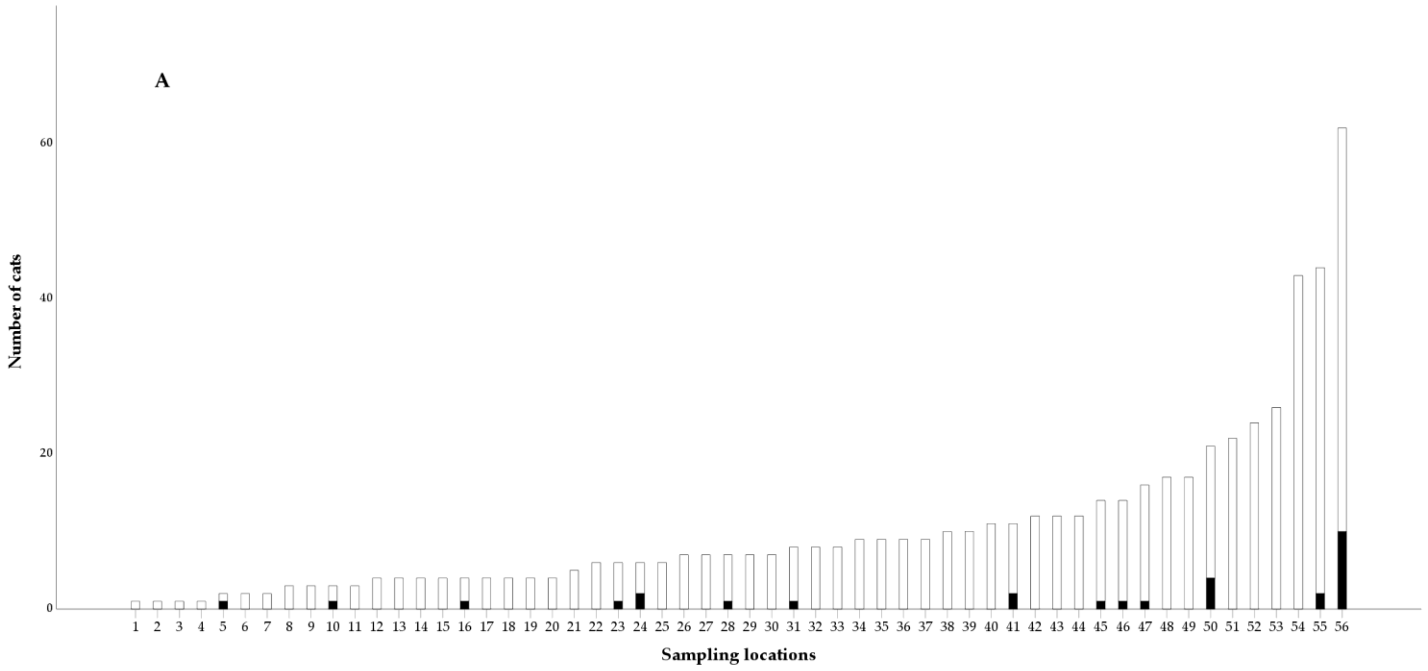
A
Number of cats
0
20
40
60
1 2 3 4 5 6 7 8 9 10 11 12 13 14 15 16 17 18 19 20 21 22 23 24 25 26 27 28 29 30 31 32 33 34 35 36 37 38 39 40 41 42 43 44 45 46 47 48 49 50 51 52 53 54 55 56
Sampling locations

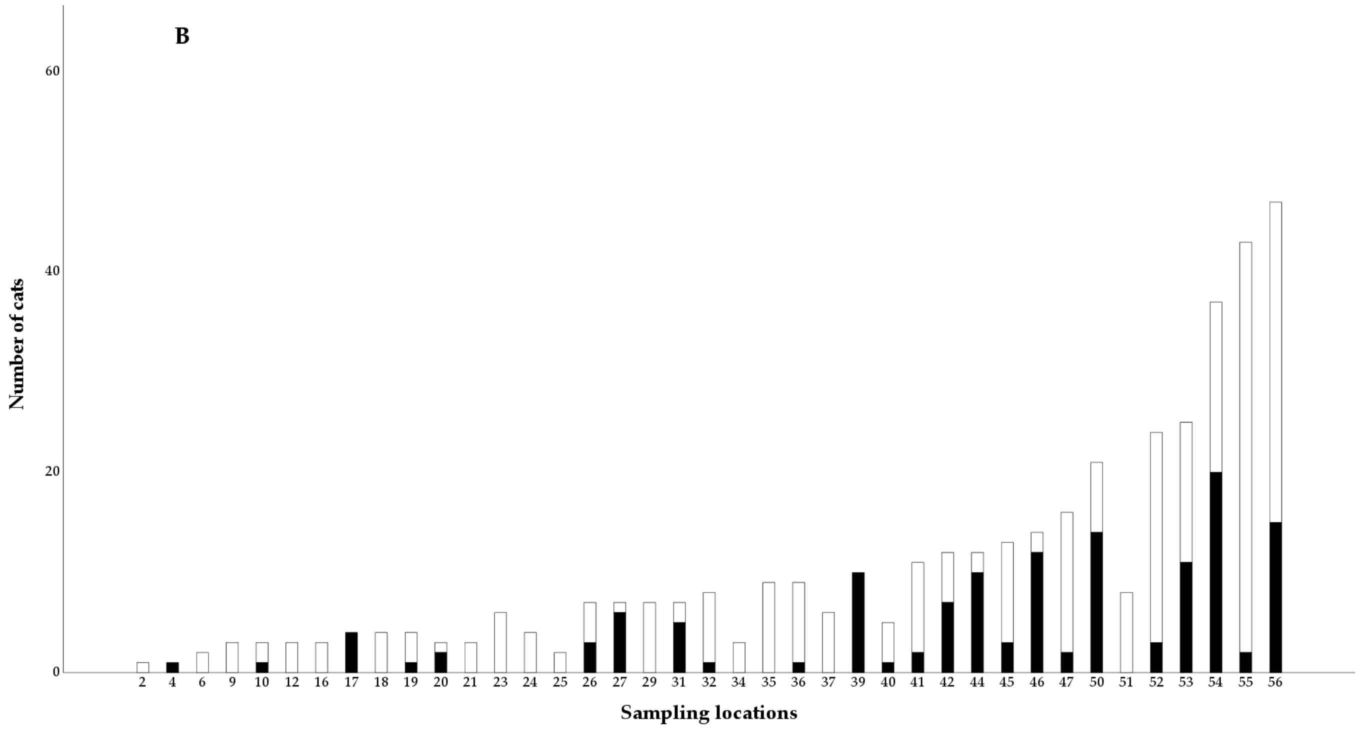
B
Number of cats
0
20
40
60
2 4 6 9 10 12 16 17 18 19 20 21 23 24 25 26 27 29 31 32 34 35 36 37 39 40 41 42 44 45 46 47 50 51 52 53 54 55 56
Sampling locations

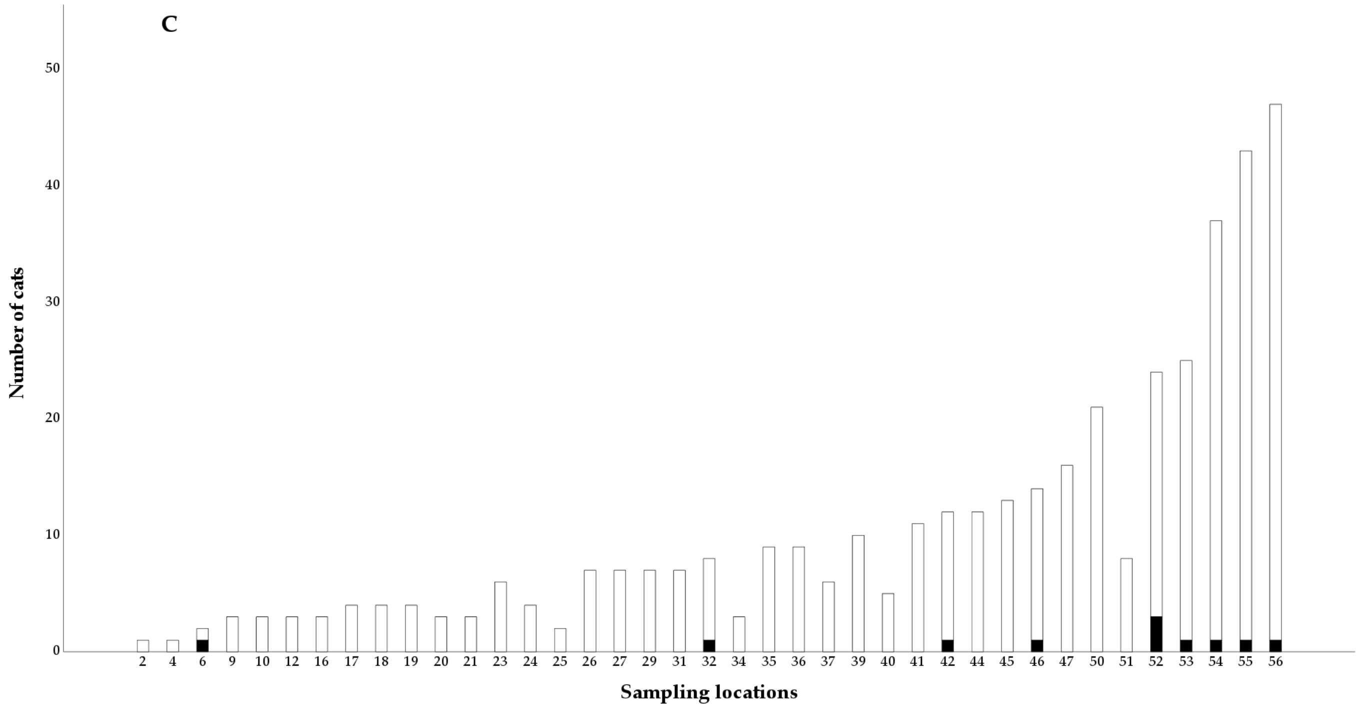

Figure S1: Number of seropositive and seronegative rural stray cats per sample location in the Netherlands, 2020-2022. A) feline immunodeficiency virus, FIV: 580 cats from 56 locations, B) feline coronavirus, FCoV: 407 cats from 39 locations, and C) severe acute respiratory coronavirus-2, SARS-CoV-2: 407 cats from 39 locations. The locations are unique and ranked based on the number of serum samples collected per location. Positive samples are shown in black and negative samples are shown in white.

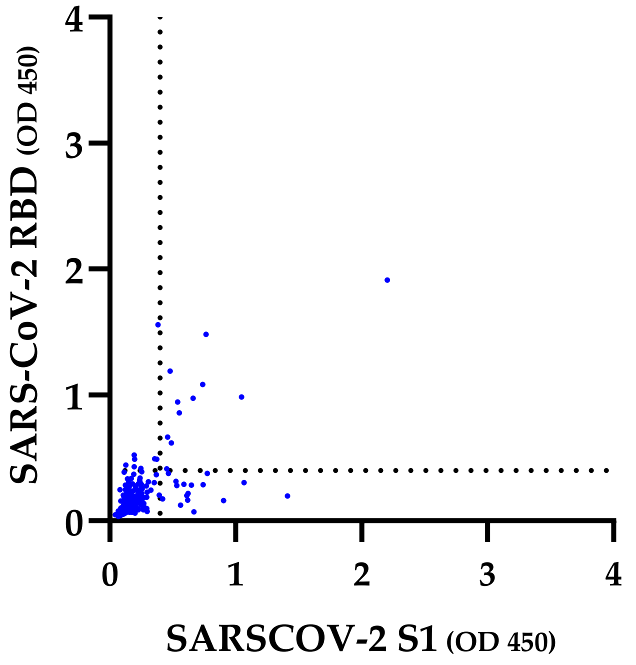

Figure S2: Correlation between the ELISA optical density of SARS-CoV-2 RBD-specific antibodies and SARS-CoV-2 S1-specific antibodies in rural stray cat serum samples (n= 407) from the Netherlands, 2020-2022. The dotted lines show the positive cut-off levels, i.e. 6-fold the standard deviation (SD) above the mean value of the spf serum sample (OD= 0.4). The number of stray cats positive in both the SARS-CoV-2 S1 and SARS-CoV-2 RBD ELISA was 11/407 (2.7%) while 371/407 (91.2%) of the stray cats were negative in both ELISA tests. The Spearman r was 0.72 (95% CI: 0.667-0.764, P-value: <0.0001).

OD: optical density measured at 450nm wavelength, ELISA: enzyme linked immunosorbent assay, SARS-CoV-2: severe acute respiratory syndrome coronavirus 2, S1: Spike protein subunit 1, RBD: receptor binding domain.

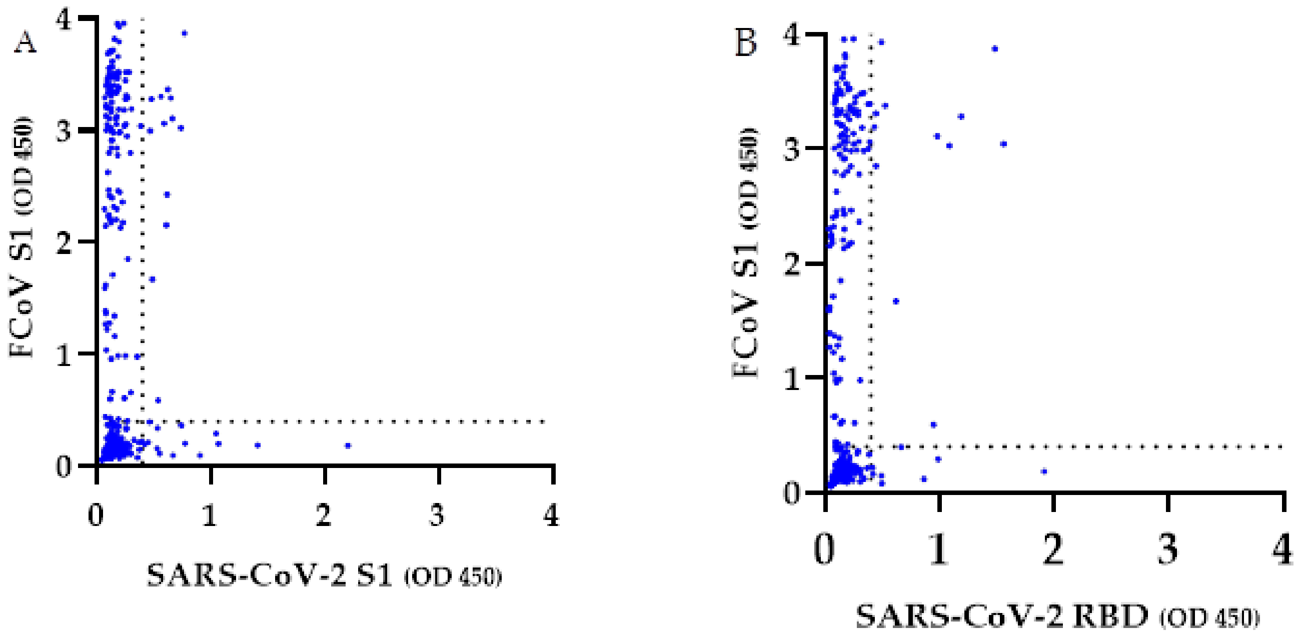

Figure S3: Comparison of the ELISA optical density of FCoV-specific antibodies and SARS-CoV-2-specific-antibodies in rural stray cat serum samples from the Netherlands, 2020-2022. A) FCoV-S1- and SARS-CoV-2 S1-specific antibodies: 13/407 (3.2%) of stray cats were positive in both the SARS-CoV-2 S1 and FCoV S1 ELISA, while 256/407 (62.9%) of the stray cats were negative in both ELISA tests. B) FCoV-S1 and SARS-CoV-2 RBD-specific antibodies: 12/407 (2.9%) of stray cats were positive in both the SARS-CoV-2 RBD and FCoV S1 ELISA, 262/407 (64.4%) of the stray cats were negative in both ELISA tests. The dotted lines show the positive cut-off levels, i.e. 6-fold the standard deviation (SD) above the mean value of the spf serum sample (OD= 0.4)

OD: optical density measured at 450nm wavelength, ELISA: enzyme linked immunosorbent assay, SARS-CoV-2: severe acute respiratory syndrome coronavirus 2, S1: Spike protein subunit 1, RBD: receptor binding domain, FCoV: feline coronavirus

Table S1. Location type and characteristics of rural stray cat sample locations in the Netherlands, 2020-2022.

**Location type**	**Cat group density ***	**Life-style**	**Human Contact #**	**Additional information**
Dairy farm	Moderate to high	Indoor (stables) and outdoor	Low	
Industrial area	Moderate to high	Indoor (storage buildings) and outdoor	Low	Sharing of litterboxes

Countryside residence	Moderate to high	Indoor (domicile) and outdoor	Low	Hoarder like situations, sharing litterboxes, feral offspring
Holiday parc or campsite	Low	Outdoor only	High	
Nature reserve	Low	Outdoor only	Low to absent	

* Cat group density was defined as moderate to high (50-100 cats per km^2) or low (<50 cats per km^2).

\# Human contact: number of human contacts was defined as low (1-5 people) or high (>10 people), none of the locations reported intermediate (>5-10) human contacts.

Table S2. Strength of association (frequencies and relative risk) of FIV seropositivity with age, sex, health status, location type, and co-seropositivity in rural stray cats sampled in the Netherlands from 2020-2022, based on locations where at least 10 cats were sampled per location.

	**Total #**		**FIV positive**		**FIV negative**				
**Variable**	**Nr.**	**%**	**Nr.**	**%**	**Nr.**	**%**	**RR**	**95%CI ***	**P-value**
**Total**	397	100	21	5.3	376	94.7		3.3-8.0	
**Estimated age**									
**<3 years**	295	76.8	5	1.7	290	98.3	Ref		
**≥3 years**	89	23.2	13	14.6	76	85.4	8.62	3.16-23.26	<0.001
**Sex**									
**Female**	222	55.9	3	1.4	219	98.6	Ref		
**Male**	175	44.1	18	10.3	157	89.7	7.63	2.28-25.64	<0.001
**Health status**									
**Apparently healthy**	262	76.8	6	2.3	256	97.7	Ref		
**Unhealthy**	91	23.2	9	9.9	82	90.1	4.31	1.58-11.77	0.004
**Location type**									
**Dairy farm**	210	55.1	14	6.7	196	93.3	Ref		0.49
**Industrial area**	10	2.6	0	0	10	100.0	0.66	0.28-1.58	
**Countryside residence**	57	15.0	4	7.0	53	93.0	1.05	0.36-3.07	
**Holiday parc/ campsite**	87	22.8	2	2.3	85	97.7	0.34	0.080-1.48	
**Nature reserve**	17	4.5	0	0	17	100	0.40	0.17-0.97	
**Co-seropositivity**									
**FeLV neg**	397	100.0	21	5.3	376	94.7			
**FeLV pos**	0	0.0	0	0.0	0				

**FCoV neg**	186	62.4	11	5.9	175	94.1	Ref		
**FCoV pos**	112	37.6	8	7.1	104	92.9	1.21	0.50-2.91	0.42
**SARS-CoV-2 neg**	289	96.9	19	6.6	270	93.4	Ref		
**SARS-CoV-2 suspected**	9	3.1	0	0	9	100.0	0.74	0.31-1.77	0.50

FeLV: feline leukemiavirus detected using antigen-ELISA, FIV: feline immunodeficiency virus detected using antibody-ELISA, FCoV: feline coronavirus detected using antibody-ELISA, SARS-CoV-2 suspected: severe acute respiratory syndrome coronavirus-2-specific antibody positive for the S1 subunit of the SARS-CoV-2 spike protein & the RBD receptor binding domain of the S1 subunit of SARS-CoV-2 detected using antibody ELISA.

Nr.: number, RR: relative Risk, CI: confidence interval, Ref: reference category.

* 95% CI Total represents the CI around the FIV seroprevalence, 95% CI for the determinants represents the CI around the Relative Risk.

# Not all serum samples were accompanied by all metadata, therefore the total numbers within a variable can be lower than N=397.

Table S3. Strength of association (frequencies and relative risk) of FCoV seropositivity with age, sex, health status, location type, and co-seropositivity in rural stray cats sampled in the Netherlands from 2020-2022, based on locations where at least 10 cats were sampled per location.

	**Total #**		**FCoV positive**		**FCoV negative**				
**Variable**	**Nr.**	**%**	**Nr.**	**%**	**Nr.**	**%**	**RR**	**95% CI ***	**P-value**
**Total**	**298**	**100**	**112**	**37.6**	**168**	**62.4**		**32.1-43.3**	
**Estimated age**									**0.021**
**<3 years**	**217**	**73.6**	**73**	**33.6**	**144**	**66.4**	**Ref**		
**≥3 years**	**78**	**26.4**	**38**	**48.7**	**40**	**51.3**	**1.45**	**1.08-1.95**	
**Sex**									**0.19**
**Female**	**164**	**55.2**	**56**	**34.1**	**108**	**65.9**	**Ref**		
**Male**	**133**	**44.8**	**56**	**42.1**	**77**	**57.9**	**1.23**	**0.92-1.66**	
**Health status**									**0.002**
**Apparently healthy**	**196**	**71**	**65**	**33.2**	**131**	**66.8**	**Ref**		
**Unhealthy**	**80**	**29**	**43**	**53.8**	**37**	**46.3**	**1.62**	**1.22-2.16**	
**Location type**									**0.017**
**Dairy farm**	**151**	**53.7**	**63**	**41.7**	**88**	**58.3**	**Ref**		
**Industrial area**	**4**	**1.4**	**1**	**25.0**	**3**	**75.0**	**0.60**	**0.11-3.31**	
**Countryside residence**	**46**	**16.4**	**25**	**54.3**	**21**	**45.7**	**1.30**	**0.94-1.80**	

**Holiday parc/ campsite**	**80**	**28.5**	**22**	**27.5**	**58**	**72.5**	**0.66**	**0.44-0.99**	
**Nature reserve**	**0**	**0**	**NA**	**NA**	**NA**	**NA**	**NA**	**NA**	
**Co-seropositivity**									
**FeLV neg**	**298**	**100.0**	**112**	**37.6**	**186**	**62.4**			
**FeLV pos**	**0**	**0.0**	**0**	**0.0**	**298**	**100.0**			
**FIV neg**	**279**	**93.6**	**104**	**37.3**	**175**	**62.7**	**Ref**		
**FIV pos**	**19**	**6.4**	**8**	**42.1**	**11**	**57.9**	**1.13**	**0.65-1.96**	**0.81**
**SARS-CoV-2 neg**	**289**	**97.0**	**106**	**36.7**	**183**	**63.3**	**Ref**		
**SARS-CoV-2 suspected**	**9**	**3.0**	**6**	**66.7**	**3**	**33.3**	**1.82**	**1.12-2.96**	**0.085**

FeLV: feline leukemiavirus detected using antigen-ELISA, FIV: feline immunodeficiency virus detected using antibody-ELISA, FCoV: feline coronavirus detected using antibody-ELISA, SARS-CoV-2 suspected: severe acute respiratory syndrome coronavirus-2-specific antibody positive for the S1 subunit of the SARS-CoV-2 spike protein & the RBD receptor binding domain of the S1 subunit of SARS-CoV-2 detected using antibody ELISA.

No: number, RR: relative Risk, CI: confidence interval, Ref: reference category, NA: not applicable.

* 95% CI Total represents the CI around the FCoV seroprevalence, 95% CI for the determinants represents the CI around the Relative Risk.

# Not all serum samples were accompanied by all metadata, therefore the total numbers within a variable can be lower than N=298.
